# Plant Sources Responsible for the Chemical Composition and Main Bioactive Properties of Poplar-Type Propolis

**DOI:** 10.3390/plants10010022

**Published:** 2020-12-24

**Authors:** Daniel Severus Dezmirean, Claudia Paşca, Adela Ramona Moise, Otilia Bobiş

**Affiliations:** 1 Faculty of Animal Science and Biotechnology, University of Animal Sciences and Veterinary Medicine Cluj-Napoca, 400372 Cluj-Napoca, Romania; ddezmirean@usamvcluj.ro (D.S.D.); claudia.pasca@usamvcluj.ro (C.P.); adela.moise@usamvcluj.ro (A.R.M.); 2 Life Science Institute, University of Agricultural Sciences and Veterinary Medicine Cluj-Napoca, 400372 Cluj-Napoca, Romania

**Keywords:** bioactive properties, chemical composition, poplar-type propolis, vegetal material

## Abstract

Propolis is a resinous mixture, made by the honeybees from substances collected from tree or other plant buds, plant exudates, or resins found in the stem, branches, or leaves of different plants. The geographical origin of propolis is given by plant sources from respective areas. Different studies have classified this bee product according to the vegetal material from the same areas. Poplar-type propolis has the widest spread in the world, in the temperate zones from Europe, Asia, or North America. The name is given by the main plant source from where the bees are collecting the resins, although other vegetal sources are present in the mentioned areas. Different *Pinus* spp., *Prunus* spp., *Acacia* spp. and also *Betula pendula, Aesculus hippocastanum,* and *Salix alba* are important sources of resins for “poplar-type” propolis. The aim of this review is to identify the vegetal material’s chemical composition and activities of plant resins and balms used by the bees to produce poplar-type propolis and to compare it with the final product from similar geographical regions. The relevance of this review is to find the similarities between the chemical composition and properties of plant sources and propolis. The latest determination methods of bioactive compounds from plants and propolis are also reviewed.

## 1. Introduction

Plants have an important role in preserving ecosystems, mostly because they are primary sources of food, as well as raw materials for industry, pharmacy, medicine, and feed for animals. Their presence influences the environment and climate, having a great impact on human lives. The biodiversity of plants also ensures resources for new pharmaceutical products and food crops.

Plants are sources of natural compounds and, in addition to humans and animals, insects find in plants the food source and the raw material for producing different natural products, which are extremely valuable for humans. This statement principally refers to honeybees and melliferous plants. Bees use plant raw materials from outside of the hive (nectar, pollen, and resins) to produce honey, bee pollen, beebread, and propolis. Honey is the main bee product, the energetic source for the bees, while bee pollen and beebread are important protein, amino acids, lipids, fatty acids, sugars, vitamins, and mineral sources for the bee family [1]. Plant resins have been used for centuries in folk medicine to treat different diseases, before discovering the antibiotics [2]. Plants, in general, and plant resins, specifically, contain a wide range of secondary metabolites with the main function of protecting the respective plants from different pathogens. The antimicrobial activity of plants is generally attributed to some of their chemical components. If these chemicals are collected, transported, and processed by the bees, definitively, a new product will be made, with the same or even better properties.

Propolis as a final product comes from the following three sources: plant resins collected by the bees; substances secreted by the bee metabolism (wax); and other materials, added by the bees during propolis elaboration [3,4]. The composition of the plant source determines the chemical composition of propolis. This depends on the specificity of the local flora at the site of collection [5]. The opinion of the European Food Safety Authority (EFSA) has been that a cause-and-effect relationship could not be established between the consumption of propolis and the claimed effects [6], because “type and content of flavonoids in propolis may vary depending on the specific propolis raw material as well as the extraction and preparation methods”.

Social insects such as honeybees live in very large groups (up to 40,000 individuals), interacting constantly, having specific roles inside the hive, and for this reason a high risk of disease and parasites may occur [7]. Bees have the ability to look for different resinous substances with antimicrobial activities, in the environment they live and collect them in, in order to protect their family and hive. Propolis serve as a natural bee health enhancer, and also as in-hive treatment. Its role in colony level immunity has been demonstrated to be more important than direct protection against pathogens and parasites [8].

For this reason, in the present review, a comparison between the plant resins as sources of bioactive compounds and the final product of the beehive, namely propolis, was made. In this review, we intend to answer the following questions: Are compounds from plant resins also present in propolis? How comparable are the bioactive properties among resins, plant extracts, and propolis? Are there synergistic or antagonistic effects between plant resins and propolis? Are plant metabolites being modified by bees? Do bee enzymes, bee microbiome, or other bee digestive substances enhance the properties of propolis? Understanding these chemical interactions will contribute to the knowledge of this important bee product and give new insights into the properties that it has and awareness for the health benefits of using it.

## 2. Plant Sources for Poplar-Type Propolis

Propolis is a resinous mixture, made by the honeybees from substances collected from tree or other plant buds, plant exudates, or resins found in the stem, branches, or leaves of different plants. These materials are generally lipophilic, such as mucilage, gums, and resins [9]. The list presented in this monograph includes numerous plant sources for propolis in different parts of the world. Two different approaches are used to determine the plant origin of propolis, i.e., observations of bee behavior or the chemical analysis of propolis and also plant materials [10]. Definitively, the second approach is more appropriate and correct, because it is scientifically proven. In early 1980s (40 years ago), scientific papers were published to evidence the similarity of plant species and propolis in different geographical regions [11,12,13,14,15,16,17].

In temperate zones, exudates from buds of the *Populus* species are the main source of resins for bees. In Europe, North America, and even New Zealand and the continental part of Australia, it has been reported that *Populus* was the main plant sources [12,13,16,17,18], although other plant resins are reported as precursors of propolis in the temperate zones of Europe and North America, including pine (*Pinus* sp.), alder (*Alnus glutinosa*), horse chestnut (*Aesculus hippocastanum*), elm (*Ulmus* sp.), ash (*Fraxinus* sp.), oak (*Quercus* sp.), and beech (*Fagus* sp.) [7,17,19,20,21]. In northern parts of Russia, aspen and silver birch buds (*Betula veruucosa*) supply bees with resins for propolis production [3,11,22,23].

Poplars (*Populus* spp., Salicaceae) include about 100 species and a lot of hybrids. These plants are the fastest growing species with very deep root systems (up to 20 m) and five-year-old trees are capable of uptaking up to 200 L of water per day [24]. Poplar hybrids, growing up to 3 m per year, are free of competition with weeds, even during the beginning of plantation.

Birches (*Betula L*.) are an essential ecological component in northern temperate and boreal forests [25]. In Europe, two important trees occur naturally, i.e., silver birch (*Betula pendula* Roth) and downy birch (*Betula pubescens* Ehrh.). These trees differ regarding the morphology of their leaves, twigs, branches, bark, seeds, and catkin scales, as well as cell size and wood anatomy, and they can reach a height of 20–30 m [26].

Generally, the genus *Salix* is very diverse, representing over 300 species [27] growing in the form of trees, shrubs, or dwarf shrubs with procumbent stems. Among the flavonoids most characteristic for poplars are flavanones, especially pinocembrin and pinostrobin. These compounds have shown antioxidant and anti-inflammatory effects in many in vitro tests and may play an important role in the pharmacological activity of *Populus* [28,29,30].

Furthermore, propolis has been used by humans as a traditional folk medicine to maintain good health since ancient times, due to many beneficial properties [31] including antioxidant, anti-inflammatory, immunomodulatory, antimicrobial, antitumor, anticancer, cardioprotective, neuroprotective, and many more [32].

Chemical determinations of propolis composition have led to the conclusion that more than one plant resin has been found in propolis [33,34,35,36] and the question raised has been if the bees show selectivity when collecting the resins in areas where multiple plant sources are found and what is the reason for this. Different studies have been published [37,38,39] that have shown that bees collect resins discriminately, due to proximity, availability, or even toxicity.

Differences in the chemical composition of poplar buds may be from different phenolic compounds such as terpenoids, flavonoid aglycones, and their chalcones, as well as phenolic acids and their esters [40], and therefore it is important to control the quality of plant material in terms of qualitative and quantitative profiles. A study conducted by de Marco et al. (2017) [4] compared the bioactive compounds of poplar buds and Italian propolis. The authors quantified the total flavonoids, chrysin, galangin, pinocembrine, and caffeic acid phenethyl ester (CAPE) that were responsible for the antioxidant activity of these matrices. The results obtained were in the range of 1.40% and 24.18% for poplar buds freeze-dried extract and 1.52% to 28.78% for Italian propolis freeze-dried extract.

Therefore, plants are the main source of bioactive compounds of propolis and bees intervene only with different enzymes to finalize the chemical composition of propolis.

## 3. Chemical Composition and Principal Properties of Plant Extracts

Genus *Populus*, commonly known as Poplars, are listed, upon the botanical method of classifying plants, as aspens and white poplars; cottonwoods and black poplars; balsam poplars; necklace poplars or bigleaf poplars; subtropical poplars and Mexican poplars, according to different databases [41]. They grow all over the world, and many of them are precursors for bee propolis. Different studies on the chemical composition of poplar buds have been conducted, and all authors have agreed that phenol carboxylic acids, their esters, flavonoids, and terpenoids were the main components of these species [42,43,44,45].

There is a regulation of the European community, stating that natural complex substances (essential oils and concentrates), used in medicine, pharmacy, or cosmetics, must be tested for their toxicity and their impact on environment, to inform consumers of risk assessment [46]. Every extract with these destinations is subjected to these determinations.

Thin layer chromatography (TLC) on silica gel and high-performance thin layer chromatography (HPTLC) RP-18 have been used in different studies for identifying and quantifying phenolic compounds from poplar bud extracts (Table 1).

As shown in Table 1, the best resolution and more identified compounds were achieved on TLC silica gel plates, using hexane-ethylacetate mobile phase for elution. The addition on an acid raised the number of identified compounds, the best acid being formic acid.

Gas chromatography-mass spectrometry (GC-MS) analysis of *P. alba*, *P. nigra,* and *P. tremuloides* bud extract from central Anatolia, were made and compared with propolis samples from the same area. The bud exudates contain aromatic acids, chalcones, flavones, flavanone, terpenes, and fatty acids [5]. This chemical profile was similar to propolis, with the only difference being the amount of these compounds. It is known that one of the propolis functions in the hive is the antibacterial activity exerted to protect the bees from different microbial invaders. Testing of the antibacterial activity of the poplar bud extracts and propolis found similar activities, mostly against Gram-positive bacteria (*Staphylococcus aureus*, *Streptococcus pyogenes*, *Listeria monocytogenes, L. innocua, L. welshimeri, and L. seeligeri*) and also against different *Candida* strains. The most effective Populus extract was obtained from *Populus nigra*, comparable to the activities exerted by propolis extract. *Populus tremula* extract had the weakest antibacterial activity [5].

In 2011, an extensive study of *Populus nigra* buds extract was conducted and different properties of the extract were also determined [40]. Raw material (*Populus nigra* buds) was extracted industrially with water at 50 °C under agitation. The solution was further concentrated and spray dried. Using HPLC for analyzing and MS and NMR for identifying, the authors reported ten phenolics (phenolic acids, i.e., caffeic, *p*-coumaric, ferulic, isoferulic, and cinnamic acid and flavonoids, i.e., pinobanksin, pinocembrin, pinobanksin 5-methyl ether, and salicin) in aqueous extract.

Due to the fact that functional foods and supplements have gained more attention during the last decades, there has been more and more demand from consumers who are preoccupied with their health to find natural sources of antioxidants. It is known that phenolic compounds possess antioxidant activity, therefore, high amounts of phenolics in different vegetal sources, suggest high antioxidant activity. The antioxidant activity of poplar buds extract was determined using oxygen radical absorption capacity (ORAC) and cellular antioxidant activity assay (CAA). Total extract of poplar buds and individually phenolics were tested for their free radical scavenging capacity. The study demonstrated that aqueous poplar bud extract, due to its phenolic composition including caffeic and *p*-coumaric acids as the major contributors, have a high antioxidant activity. Caffeic acid also has high antioxidant activity in cellular antioxidant activity assay determination [40].

Another study on *Populus nigra* bud extracts was conducted to determine their chemical composition [50]. Using vacuum molecular distillation, three different fractions were obtained (a volatile fraction, a distilled fraction, and a residue fraction) and analyzed afterwards by gas chromatography with flame ionization detection (GC-FID-MS), gas chromatography mass spectrometry (GC-MS), and high-performance liquid chromatography-diode array detection. From the fractions obtained after distillation, 91 compounds were identified after derivatization and GC-FID-MS analysis and 52 compounds through GC-FID-MS analysis without derivatization. Among these compounds, phenolic acids, flavonoids, alcohols, and terpenoids were identified. Thirty phenolic acids, flavonoids, and also caffeic acid phenyl-etil ester (CAPE) were identified through high performance liquid chromatography diode array detection, electron spray ionization mass spectrometry (HPLC-PDA-ESI-MS) analysis. High amounts of *p*-coumaric acid; 1, 1-dimethylallyl caffeate; pinostrobin; pinocembrin; chrysin; and galangin were determined (1.26–5.32%) [51]. These compounds are also major components in poplar-type propolis [50,52,53,54,55]. Other different studies have shown that *Populus nigra* buds also present high amounts of terpenoids and phenolic compounds [43,56,57]. Jerković and Mastelić (2003) [57] elucidated the volatile composition of poplar buds (*Populus nigra* L.) and connected it with the chemical composition of propolis, used a simultaneous hydrodistillation-extraction procedure to obtain essential oil. The analyses were made using GC/MS procedure. Different classes of terpenes were identified (hemiterpenes, monoterpenes, sesquiterpene hydrocarbons, sesquiterpene alcohols, and other acids and alcohols), the total yield being 0.27% from fresh buds and 0.12% from dried buds. Differences were observed in the pattern of compounds in *Populus nigra* and other *Poplar* species [58].

High amounts of phenolics have been determined in bud extracts of *Populus nigra* from Algeria [59]. The antioxidant activity of the extracts was tested using DPPH scavenging activity; ABTS; and H_2_O_2_ scavenging activity; OH·, HOCl·, and NO scavenging activity; inhibition of lipid peroxidation; and anti-inflammatory activity (inhibition of xanthine oxidase and carrageenan-induced paw edema). The biological activity of all vegetal extracts resides from the bioavailability of the phytochemicals in the organism. Caffeic acid and quercetin, important phenolic compounds present in *Populus nigra* extracts, are responsible for the high biological activity of poplar-type propolis.

**Figure 1:**
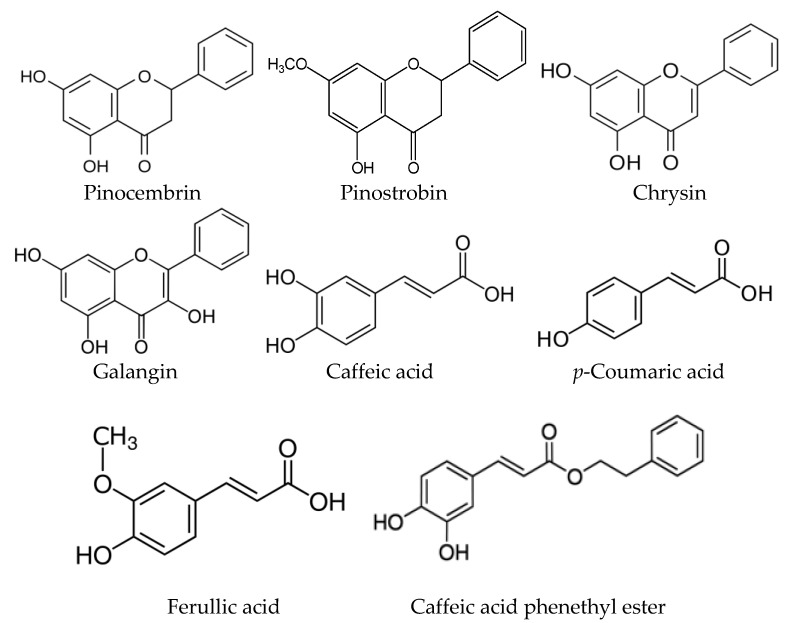
Chemical structures of the most important polyphenolic compounds of *Populus* bud extracts.

Many available studies have revealed the bioactive properties of *Populus* bud extracts (*P. nigra*, *P. balsamifera*, *P. tremula*, *P. canadensis,* and their hybrids) [40,60,61,62,63,64]. *Populus* taxa can be divided in two important groups with respect to their chemical composition, i.e., one group having flavonoids as the major components and another group having phenolic acids and their derivatives as major components [60]. From the most important compounds of polyphenolics (flavonoids and phenolic acids), *Poplar* bud extracts contain pinocembrin, pinostrobin, chrysin, galangin, caffeic acid, *p*-coumaric acid, and caffeic acid phenethyl ester (Figure 1). These compounds play a very important role in the determination of bioactive properties of *Populus* extracts [28,29,63].

The study of Falcão et al. (2016) [65], on volatiles from essential oil of *Poplar* x *canadensis*, and propolis from the same region, indicated that the botanical origin of the plant was the determinant of propolis composition and properties.

One of the latest studies by [30], tested the anti-inflammatory and antioxidative effects of different *Populus* species bud extracts in human gingival fibroblast cells. The methanolic extracts of different varieties of *Populus* were characterized, and flavonoid quantification was made with TLC determinations. Next, a free radical scavenging activity (DPPH method) and a riboflavin-light-NBT test were used to determine the antioxidant activity of the extracts. Another experiment was the bioautographic assay of xanthine oxidase (XO) inhibition [66]. Inspired by previous studies [67], the authors tested poplar extract on HGF-1 cells, treated with AgNPs (silver nanoparticles used in dental practice, being constituents in dental implants, endodontic retrofill cements, or different restorative materials), which caused inflammation. The HGF-1 cells were treated with pinocembrin, pinostrobin, and poplar bud extract in different concentrations. A 3-(4, 5-dimethylthiazolyl-2)-2, 5-diphenyltetrazolium bromide (MTT) test for viability was conducted and, afterwards, the levels of interleukins (IL-6 and IL-1β) were determined. Statistically significant downregulation of RNA transcript levels of IL-6 and IL-1β in HGF-1 cells treated with the flavonoids, as well as the extract, were observed as compared with the control (cells exposed only to silver nanoparticles AgNPs). The experiment showed that poplar extract (and simple flavonoids contained in the extract) reduced the level of the proinflammatory interleukins IL-6 and IL-1β, in a concentration dependent way [68]. Knowing all these biological activities and the chemical profile of poplar bud extracts, similar properties are expected from poplar-type propolis.

## 4. Poplar-Type Propolis

Propolis is a bee product, made by the honeybees (*Apis mellifera*) from different resins, collected from plant leaves, buds, or exudates, mixed with bee saliva and wax [69]. This mixture is taken into the hive and used to protect the bee family from outside enemies, to bond the frames between them, to seal any hole in the hive, and to maintain a stable indoor temperature [21]. The color of propolis varies greatly with the botanical source and geographical origin. Poplar-type propolis color can vary from yellow orangish, to reddish and brown, or dark brown. Plant bud resins from *Poplar* species are primary sources for propolis from temperate zones (Europe, North America, and Asia), but also other species contribute to the chemistry of propolis from these areas (*Betula* sp., *Acacia* sp., *Pinus* sp., *Salix,* or *Aesculus hippocastanum* [70]. To the best of our knowledge, poplar type propolis have the widest spread along the globe, and its composition and properties are the most studied from all bee products, apart from honey.

Having such a large distribution over the temperate zones of the globe, poplar-type propolis is also very different in chemical compounds, although volatiles from the class of terpenoids and polyphenolic substances (phenolic acids and flavonoids) are the major compounds. A recent study by [71], identified a new type of propolis rich in flavonoids which exhibit also a very powerful antibacterial activity.

Different studies on the chemical composition of propolis have made possible the classification of propolis from different countries, knowing the fact that European propolis have as the main vegetal source, the exudates from different *Populus* species. It is generally accepted that propolis from temperate zones are rich in pinocembrin, pinobanksin, galangin, chrysin, caffeic, and ferulic acids; these are all phenolics reported in *Poplar* exudates. Miguel (2013) [53] reviewed the propolis type of countries from the Mediterranean basin. Italian propolis samples analysis revealed the presence of phenolic acids and flavonoids as the main components and concluded that poplar-type propolis was characteristic to Italy [72,73]. Other studies [74,75] have reported also phenolic acids and their esters and flavonoids in propolis samples from France. Hydroalcoholic extracts of propolis from Spain, revealed the presence of flavonoids as predominant components, demonstrating the poplar appurtenance [76,77]. Portuguese propolis characterization revealed methylated and/or esterified or hydroxylated derivatives of poplar flavonoids [53,78,79].

### 4.1. Chemical Composition and Analysis Methods

Propolis is known to be a very important natural antibiotic. Its properties were observed before its chemical composition was really analyzed. Before the development of separation and purification techniques to reveal chemical components of propolis, the existing studies focused mostly on bioactive properties and mainly attributed its entire composition to these properties. After the mentioned techniques were used more and more, the chemical composition of propolis was established, and its properties were attributed to different classes of compounds originating from different geographical areas [42,53,55,78,80,81,82].

Generally speaking, poplar-type propolis have about 50% resins, 30% beeswax, 10% aromatic oils, 5% pollen, and 5% other substances (minerals, vitamins, and amino acids) [18], and, so far, more than 350 compounds have been identified and quantified [83,84].

Different scientific studies have classified these components as phenolic acids and their esters, all classes of flavonoids (aglycones and glycosides), chalcones and dihydrochalcones, terpenes and hydrocarbons, alcohols and their esters, aldehydes, amino acids, fatty acids, sterols, sugars, and sugar alcohols [83]. The majority of these substances came from resins, plant exudates, but also from bee metabolism. Sugars and pollen came from cross contamination with nectar and the fatty component of propolis (fatty acids, esters, and glycerol) came from beeswax [83]. The major compounds of poplar-type propolis all over the world are presented in Table 2. As can be seen in the table, the majority of compounds belong to polyphenolic substances.

Over time, the methods used for propolis analysis have evolved significantly. Due to the nature of the main components of propolis, spectrophotometric and chromatographic (liquid and gas) methods have been used. Two different types of extraction are used in propolis analysis, i.e., extraction for the nonvolatile metabolites and the extraction for volatiles analysis. The first class of compounds are obtained by simple extraction with ethanol or methanol of different concentrations, extraction time, and temperatures [22]. Because no international regulations are available for propolis analysis, different conditions are used for these extractions. Generally, phenolic compounds are determined by liquid chromatography with different detections. A study on Portuguese propolis [79] used liquid chromatography with diode-array detection coupled to electrospray ionization tandem mass spectrometry (LC-DAD-ESI-MS) and characterized the phenolic compounds by comparing UV spectra, retention time, and MS information (*m/z* values) with reference compounds.

The most recent study on propolis phenolics and volatiles [98] used ultrahigh-performance liquid chromatography with diode array detector and quadrupole time-of-flight mass spectrometry (UHPLC-DAD-QqTOF-MS), and identified a high number of compounds (118 phenolics), suggesting that equipment and methods that are more elaborate and up-to-date can identify and quantify more compounds.

Propolis volatiles are responsible for the aroma and smells of the product, although they are found in small concentrations. Volatiles may give important information regarding plant sources, and thus the origin of propolis. Volatiles that present as the most abundant compounds in poplar-type propolis, include monoterpenes, sesquiterpenes, and organic compounds [10,43,85,101].

The most important criteria in gas chromatography mass spectrometry analysis is the computed match factor of the spectrum and the respective one in the existing library [100]. The identification of the compounds is generally done by computer searches in available libraries. In GC analysis, in some cases, unidentified compounds remain, because their spectra are not found in the respective libraries. In these cases, only the structural type of the compound is proposed, based on the fragmentation spectrum of the query compound.

Another method of propolis composition analysis is fourier transform infrared attenuated total reflectance (FTIR-ATR) [98]. The complexity of the propolis spectrum measured by FTIR, give its’ overall chemical composition, and the identification of every signal represents a demanding task. Trained specialists can distinguish different signals corresponding to particular organic compounds, based on the literature data of propolis composition and different spectral data of FTIR libraries. The mentioned study is among the few studies existing on propolis analysis.

Over the last decades, the old method of TLC has been improved, and coupled with high-performance liquid chromatography, for direct identification of the antioxidant compounds of poplar propolis and other natural matrices, using also antioxidant radical 2, 2-diphenyl-1-picrylhidrazyl (DPPH) [105,113]. The method is based on the separation of bioactive constituents from the polyphenolic class using high-performance thin layer chromatography, visualization of the compounds being made using DPPH as the derivatizing reagent. Overall, the most used analysis methods for chemical composition of propolis extracts remains liquid and gas chromatography.

### 4.2. Main Bioactive Properties of Propolis

Regarding the bioactive properties of propolis, there are many demonstrated activities such as antioxidant [114,115,116,117], anti-inflammatory [118], antibacterial [119,120,121,122], antifungal [123,124,125], anticancer [126,127,128,129], immunosuppressant [118], and antiviral activity [130,131,132,133,134,135]. Antioxidant activity of propolis extracts have been evaluated over time using different spectrophotometric methods in vitro. The simplest method used for antioxidant activity determination for different natural extracts, including plants and in our case propolis, is radical scavenging activity (RSA) using 2, 2-diphenyl-1-picrylhidrazyl (DPPH) assay. DPPH is a stable radical which reacts with bioactive compounds present in the extract and is expressed as a % of inhibition [136,137]. Combining HPTLC and DPPH, a new method has been developed that is simple and accurate, which facilitates explorative work by testing different natural matrices with complex chemical composition [113]. The method is regarded as a novel analytical quality control tool that can be applied to different complex natural matrices. Ferric reducing ability power (FRAP) assay is based on the redox reaction between the bioactive compounds contained in the extract and the Fe^3+^-TPTZ complex (FRAP reagent) and is expressed as the potential of the antioxidants to reduce Fe^3+^ to Fe^2+^, which is spectrophotometrically measured at 593 nm [138].

Miguel et al. [115] demonstrated that there were no statistical difference between the antioxidant activities of brown propolis harvested in different seasons of the year. According to their results, the ABTS (2, 2’-azino-bis (3-ethylbenzothiazoline-6-sulfonic acid) value was between 0.019 mg/mL in spring collected propolis and 0.020 mg/mL in those harvested during the winter period. The DPPH values ranged between 0.027 mg/mL and 0.031 mg/mL, respectively.

A more recent study by Seibert et al., 2019 [117] reported that the concentration required to obtain a 50% antioxidant effect of propolis (EC50) using the DPPH method was 25.04 for ethanolic extracts and 3.14% when the ABTS method was applied. For hexanic extracts and etyl acetate extracts, with both methods, the values were superior.

Svečnjak et al. (2020) [98] analyzed seven raw propolis coming from the Croatian Islands and stated that the highest activity was observed for the samples of *Populus* spp. origin. The antioxidant potential of these samples, determined by DPPH ranging from 2.6 to 81.6 mg GAE/g and by FRAP assay values ranging from 0.1 to 0.8 mmol Fe^2+^/g were registered.

Generally, in propolis research observations, the antimicrobial activity of the extracts has been higher in Gram-positive as compared with Gram-negative bacteria, where limited effects have been observed [139,140,141]. Gram-negative bacteria have a species-specific structure of the outer membrane and produce a hydrolytic enzyme which breaks down the active ingredients of propolis [142]. The antibacterial activity of propolis is due to its bioactive compounds (aromatic compounds and polyphenols). Interactions among different classes of chemical compounds have an important role, which has also been demonstrated against *Paenibacillus larvae* (a honeybee pathogen) [143]. Flavone/flavonols and flavanone/dihydroflavonols are the two main classes of phenolics in propolis. The mentioned study developed a statistical model to detect a potential interaction between the two classes of flavonoids and the inhibition activity of different propolis extracts (10 mg/mL) originating in different geographical origins from Romania on *Paenibacillus larvae*. The inhibitory effect of different propolis extracts was statistically significant. The content of these compounds influences the strength of antibacterial effects, and the significant interaction effect between flavonoids should also be taken into consideration. How does propolis acts as bactericidal agent? It has a direct action on the microorganism and another indirect activity by stimulating the immune system of the bees for activating natural defense of the organism against different bacterial diseases. The process stops the division of bacterial cells, destroying the cell wall and bacterial cytoplasm, and thus stopping the bacterial protein synthesis, as described in different scientific studies [144,145,146,147].

A comprehensive review was published recently [148] that characterized the latest studies on the antibacterial activity of propolis on Gram-positive (*Staphylococcus aureus*, *S. epidermidis*, *Streptococcus mutans*, *S. viridians*, *S. pyogenes*, *S. pneumoniae*, *S. oralis*, *S. agalactiae*, *S. sobrinus*, *Enterococcus* spp. *Micrococcus luteus*, *Bacillus subtilis*, and *Clostridium dificile*) and Gram-negative bacteria (*Escherichia coli*, *Salmonella* spp., *Klebsiella* spp., *Yersinia enterocolitica*, *Proteus mirabilis*, *Shigella flexneri*, *Enterobacter cloacae*, *Enterobacter aerogenes*, *Pseudomonas aeruginosa*, *Acinetobacter baumannii*, *Haemophilus influenza*, *Campylobacter jejuni*, *Bacteroides fragilis*, and *Burkholderia cepacia*). Propolis antibacterial activity was most often tested on *E. coli*, *S. aureus*, *Salmonella* spp, and *P. aeruginosa* [148]. More than 600 bacterial strains were tested, according to literature studies, and the efficacy of propolis on Gram-positive over Gram-negative bacteria was confirmed, the first class presenting lower minimum inhibitory concentrations (MIC) over the second class [148].

Cancer is one of the most severe and often deadly diseases in our times. Treatment methods include surgery, but also chemotherapy, radiotherapy, or immunotherapy according to individual characteristics of the patient. Chemotherapy and radiotherapy have different toxic effects and, nowadays, different antioxidant substances are used as enhancers of the immune system and reduce the toxic effects on patients. Propolis is a very powerful antioxidant and its antiproliferative activity has been tested either in vitro on different cancer cells or in vivo on animal models, where reduction of the tumor was observed. In 2003, Orsolic and Basic [149] observed an anti-metastatic activity of a water-soluble propolis derivative upon a CBA mouse mammary carcinoma tumor. The propolis derivative reduced the metastases in mice lung and also changed several immunological parameters of mice. Other different malignant cells (ME45 malignant melanoma, HTC 116, Caco-2, DLD-1, HT-29 human colorectal carcinoma, A549 and H23 lung cancer cells, MCF-7 hormone dependent and MDA-MB-468 human breast cell lines, LN18, and U87 glioblastoma cell lines), were treated in vitro with propolis extracts and an antitumor activity was observed, dependent on the cell lines. At the same time, L-929 normal fibroblast cells were not affected by propolis at a concentration of 1 μg/mL [103]. A recently published study [150] used propolis and a new designed product (chitosan-coated nano-propolis NP) to reduce the side effects of cisplatin, a drug widely used in cancer treatment. The in vivo study used Wistar rats divided into seven groups with different treatment schemes. The experimental groups treated with propolis and NP ameliorated the cisplatin effect and protected liver and kidney tissue from the toxicity induced by the drug.

Another important property of propolis extracts is exerted in oral cavity diseases [139]. Dental caries can be caused by different bacteria (*Streptococcus mutans*, *S. sobrinus*, different *Actinomyces,* and *Lactobacillus*). Propolis extract have antimicrobial activity against *L. fermentum* isolated from cavities of patients diagnosed with dental caries [120]. A comprehensive review on the potential uses of propolis in oral health was published in 2010 by Parolia et al. [144]. Different beneficial properties of propolis were mentioned, which included dental surgical wound healing [151], new storage media following avulsion [152,153,154], pulp capping agent [155], as an intracanal irrigant [156], as a mouth rinse [153,157], for dentinal hypersensitivity [158,159], for treatment of perodontitis [160], for treatment of denture stomatitis [161], as an intra-canal medicament [162], an effect on recurrent aphthous stomatitis [163], and an effect on *Candida albicans* [164]. A conclusion of the review was that propolis can be used in all these pathologies, but cautions must be taken due to some allergic reactions in some patients. Propolis extracts can also be used in the composition of mouthwashes and toothpastes, to enhance the prevention of microbial infection and treatment of gums inflammation [147].

## 5. Propolis and New Trends in Medicine: Antiviral Activity

Viral diseases represent a very serious issue for public health. Humans have been faced with different viral epidemics over the last decades. The most contagious were severe acute respiratory syndrome coronavirus (SARS-CoV), in 2002 and 2003, which was a viral disease caused by the H1N1 virus (2009) and the Middle East respiratory syndrome coronavirus (MERS-CoV), in 2012.

The latest and the novel epidemic, observed and reported in the last months of 2019 and continued in 2020, was a “new” coronavirus spread all over the world and causing “COVID-19” disease [165,166,167,168].

Many studies have discussed the very important activity of propolis, i.e., antiviral activity, in the last months, regarding its use during the COVID-19 pandemic. The antimicrobial and especially antiviral properties of propolis against herpes simplex virus, influenza virus, avian influenza virus, HIV, parvovirus, adenoviruses, and many others, have been well documented [123,131,132,133,134,169,170,171,172,173,174,175,176]. These activities are due to the chemical composition of propolis, which include different classes of phenolics and terpenes. The main flavonoids responsible for the antiviral activity of propolis are quercetin, pinocembrin, kaempferol, myricetin, and caffeic acid and its derivatives [82,177,178,179,180,181,182,183], which reduce or even block the entrance of the virus into host cells [184]. This process is considered to be an early step of the viral replication; using propolis (with the mentioned phenolic compounds in the chemical composition) may help and be suitable for prophylaxis by stimulating the adaptive immune response of the host [185].

Problems with COVID-19 infections in humans are represented by the comorbidities. Different metabolic diseases are considered to be comorbidities (cancer, cardiovascular diseases, diabetes, kidney diseases, obesity, and senescence) [186,187,188,189]. Due to a weak immune response by the host, any viral disease that is already known as highly contagious may be contacted more easily.

Where could propolis interfere in this problem? For some time, different studies have demonstrated the role of propolis as especially that of enforcing the organism immune system [135,184,189]. Polyphenolic substances (the main chemical components of propolis) are powerful antioxidants, capable of scavenging free radicals and protecting the cell membrane against lipid peroxidation, and therefore enforcing the immune system.

As far as it is published, there is a randomized in vivo study conducted in Brazil, to evaluate the effects of Brazilian green propolis extract on patients with COVID-19, on oxygen therapy duration and total hospitalization time [190]. Another Brazilian group of experts [191] have been studying propolis in conjunction with different SARS, MERS and other viruses. The results are also very promising in the recent SARS-CoV-2 pandemic.

The low cost, safety of the product, health promising effects, and ease of administion, make propolis an important ingredient in the prevention and supporting therapy for SARS-CoV-2, as it has been for other viruses and viral diseases.

## 6. Conclusions

Propolis is a very important bee product for bees and also humans. It has a broad chemical composition, and it is composed of an impressive number of bioactive substances, giving it special therapeutic properties. Plant exudates, used by the bees as sources for propolis production, have a similar composition and properties as propolis from the same areas. Starting from the plant sources of poplar-type propolis and their chemical composition, this review synthetizes its chemical composition and bioactive properties, demonstrating that the resins’ chemical composition has a decisive role on the final product, and that the contribution of bees on its final properties is smaller. Bees do not change the substances that are collected from the plant buds, but they enrich them with their own substances, enhancing, in this way, the exerted properties. Scientific studies have demonstrated that in propolis extracts the bioactive capacity are higher as compared with exudate extracts. Generally, propolis is considered to be an animal product, but due to the minimal contribution from bees, it remains to be an “herbal drug”. This review highlights the chemical composition and bioactive properties of propolis, and its importance in treatments for a number of human diseases. For the extensive use, of propolis as a supplement or drug, further research is required in order to standardize this valuable product. Although such research is difficult, due to the fact that its botanical origin is different and the harvest season influences its chemical composition as well as the extraction process, we recommend that validated control methods should be developed for making the use of this product more efficient.

## Figures and Tables

**Table 1:** Thin layer chromatography (TLC) analysis of phenolic compounds in extracts from different poplar species.

Plant Material	Separated Compounds	Adsorbent/Mobile Phases	Reference
Brazilian poplar buds	Flavonoid profiles	HPTLC RP-18 F_254_Merck/Ethanol-water (55:54, *v*/*v*)	[47]
Brazilian poplar buds	Gallic, ferulic, caffeic *p*-coumaric acids, quercetin, kaempferol, chrysin, pinocembrin, pinostrobin	TLC Silica gel 60 Merck/Hexane-ethyl acetate (3:2, *v/v*)	[48]
*Populus balsamifera*	Neutral substances (acylglycerides and sterols)	TLC Silica gel L40/100/petroleum ether-diethyl ether-acetic acid (80:20:1, *v/v/v* or 70:30:1, *v*/*v*/*v*); heptanes-benzene (9:1, *v/v*)	[49]
*Populus nigra*, *P. nigra* “Italica”. *P*.x *can*.”Robusta”, *P*.x *canescens*, *P*. *berolinensis*, *P*. *maximowiczii*, *P*. *balsamifera*, *P*. *tremula*	Apigenin, quercetin, kaempferol, chrysin, naringenin, caffeic acid phenethylester (CAPE), galangin, pinocembrin, caffeic acid	TLC Silica gel 60 Merck/hexane-ethylacetate-glacial acetic acid (5:3:1, *v/v/v*)	[50]
*Populus alba*, *P*. *tremula*, *P*. *nigra* “Italica”, *P.* x *canadensis* “Robusta”, *P*. *canadensis* “Marilandica”, *P*. *balsamifera*, *P*. *candicans*, *P*. *simonii*	Apigenin, luteolin, genkwanin, chrysin, tectochrysin, galangin, isorhamnetin, kaempferol, quercetin, myricetin, eriodictyol, naringenin, pinocembrin, pinostrobin, pinobanksin, chrysin 5, 7-dimethylether, pinocembrin 5, 7-dimethylether	TLC Silica gel 60 Merck/hexane-ethyl acetate-formic acid (60:40:1.3, *v/v/v*)	[44]

**Table 2:** Chemical composition of poplar-type propolis of the major producing poplar-type propolis of the world.

Origin	Separated Compounds	Analytical Method	Reference
Portugal	Caffeic acid, ellagic acid, *p*-coumaric acid, ferulic and isoferulic acid, quercetin, luteolic, apigenin, kaempherol, rhamnetin, chrysin, galangin, acacetin, kaempferide, kaempferol dimethyl ether, other flavonoid glycosides, sesquiterpene, monoterpene, aliphatic and aromatic alcohols, fatty acids, carbonyl compounds, hydroxarbons	LC-DAD ESI-MS; GC-MS	[65,78,79,85]
Italy	Communic acid, isocupressic acid, acetylisocupressic acid, caffeic acid, *p*-coumaric acid, ferulic acid, quercetin, apigenin, kaempferol, chrysin, caffeic acid phenethyl ester, pinocembrin, galangin, benzyl salycilate, benzyl cinnamate, caffedic acid cinnamyl ester, pinobanksin-3-O-acetate	GC-MS, HPLC-MS/MS	[32,72,73,86]
Spain	Naringenin, genistein, kaempferol, apigenin, pinocembrin, galangin, acacetin, chrysin, benzoic acid, guaiol, pinostrobin, pinobanksin, galangin-7-methyl ether, pinobanksin-3-acetate, glyceryl trans-caffeate, henicosane, tricosane, pentacosane, hexacosane, heptacosane, nonacosane, tetracosanoic acid	GC-MS, HPLC-ESI-MS	[71,76,87]
France	Benzyl caffeate, pinocembrin, trans-*p*-coumaric acid, caffeic acid, *p*-coumaric acid, chrysin, pinobanksin, pinobanksin-3-acetate, galangin, kaempferol, tectochrysin	GC-MS, RP-HPTLC-FLD, RP-HPTLC-DART-MS	[74,88]
Bulgaria	Dihydrocaffeic acid, dihydroferulic acid, pinostrobin, dimethyl kaempferil, benzyl alcohol pinobanksin, chlorogenic acid, caffeic acid, *p*-coumaric acid, ferulic acid, quercetin, myricetin, kaempferol, rutin, catechin, quercetin-3-*β*-glucside, alcohols, aromatic acids, organic acids, terpenoids	HPLC, GC-MS	[89,90,91]
Turkey	Apigenin, pinocembrin, pinobanksin, chrysin, galangin, quercetin, rutin, kaempferol, *p*-coumaric acid, ferulic acid, caffeic acid and their esters, *p*-hidroxybenzoic acid, vanillic acid, protocatechuic acid, cinnamyl cinamate, abietic acid, isopimaric acid, dihydroabietic acid, hydroxy fatty acids, phenolic glicerides	TLC; HPTLC, GC-MS; UHPLC-LTQ/Orbitrap/MS/MS	[92,93,94,95]
Romania	Gallic acid, protocatechuic acid, syringic acid, caffeic acid, vanillin, *p*-coumaric acid, ferulic acid, *t*-cinnamic acid, rosmarinic acid, pinocembrin, chrysin, galangin, pinostrobin, caffeic acid phenethyl ester, rutin, quercetin, apigenin, resveratrol	HPLC-DAD, LC-MS	[52,96,97]
Croatia (islands in Mediterranean Sea)	108 Volatiles determined by HS-SPME/GC-MS 118 Compounds determined by UHPLC-DAD-QqTOF-MS (terpenes, phenolic acids, flavonoids and their derivatives)	GC-MS, FT-MIR, UHPLC-DAD-QqTOF-MS	[98]
Greece	59 Phenolic compounds determined by HPLC-PDA-ESI/MS	HPLC-PDA-ESI/MS, GC-MS	[99,100,101]
Poland	Aromatic acids, fatty acids, esters, flavonoids, and chalcones (85 constituents) 37 Phenolic compounds identified by LC-MS	GC-MS, HPLC-DAD, LC-MS, UPLC-Q-TOF-MS	[102,103,104]
Germany	Chrysin, pinocembrin, naringenin, pinobanksin, kaempferol, luteolin, pinobanksin-5-methylether, coumaric acid, galangin, apigenin, pinostrobin, benzyl caffeate	HPTLC	[105]
Morocco	Wogonoside, quercetin-arabinoseglucoside, apigenin dihexoside, rhamnetin hexoside, baicalin, rhamnetin, isorhamnetin, saphnin, daphnitin, afzelechin-catechin dimmer	HPLC-ESI/MS	[106]
Algeria	Pinostrobin chalcone, galangin, naringenic, tectochrysin, methoxychrisin, prenitated coumarin, pectolinaringenin, pilosin, ladanein, chicoric acid, caftaric acid, 2-hexanal, myristic acid, linoleic acid, spathulenol, isooctgane, hexadecane, *p*-cymene, palnitic acid, 4-terpineol, charvacol, α-cedrol	Two-dimensional paper chromatography, identification by ^1^H NMR and ^13^C NMR, HPLC-DAD, GC-MS	[107,108,109,110]
Tunisia	Chrysin, galangin, tectochrysin, pinocembrin, pinobanksin, dimerthyallil caffeate, phenylethyl caffeate, myricetin 3, 7, 4′, 5′-tetramethyl ether, quercetin 3, 7, 3′-trimethyl ether	HPLC	[111]
Canada	Chrysin, pinocembrin, ellagic acid, pinostrobin, benzyl caffeate, palmitic acid, naringenin, pinobanksin, isopentenyl caffeate, acacetin, caffeic acid, acacetin, caffeic acid phenethyl ester, other aromatic acids, fatty acids, esters, dihydrochalcones	HPLC-ESI/MS, GC-MS	[16,80,112]

LC-DAD ESI-MS, liquid chromatography diode array detection, electron spray ionization mass spectrometry; GC-MS, gas chromatography-mass spectrometry; HPLC-MS/MS, high-performance liquid chromatography mass spectrometry/mass spectrometry detection; RP-HPTLC-FLD, reverse phase high-performance thin layer chromatography fluorescence detection; RP-HPTLC-DART-MS, reverse phase high-performance thin layer chromatography direct analysis in real time mass spectrometry; HPLC, high-performance liquid chromatography; TLC, thin layer chromatography; HPTLC, high-performance thin layer chromatography; UHPLC-LTQ/Orbitrap/MS/MS, ultrahigh-performance liquid chromatography linear trap quadrupole and Orbitrap mass spectrometry/mass spectrometry detection; HPLC-DAD, high-performance liquid chromatography diode array detection; LC-MS, liquid chromatography mass spectrometry; FT-MIR, Fourier transformed mid infrared detection; UHPLC-DAD-Qq-TOF-MS, ultrahigh-performance liquid chromatography diode array detector and quadrupole time-of-flight mass spectrometry; UPLC-Q-TOF-MS, ultraperformance liquid chromatography quadrupole-time of flight mass spectrometry; ^1^H NMR, proton nuclear magnetic resonance; ^13^C NMR, carbon 13 nuclear magnetic resonance.
